# Decoding thermal resilience in fish: acute warming tolerance is associated with neural failure in rainbow trout

**DOI:** 10.1098/rsbl.2025.0132

**Published:** 2025-07-30

**Authors:** Andreas Ekström, Irena Senčić, Jeroen Brijs, Albin Gräns, Erik Sandblom

**Affiliations:** ^1^ Department of Biological and Environmental Sciences, University of Gothenburg, Gothenburg, Sweden; ^2^ University of Innsbruck, Innsbruck, Austria; ^3^ Department of Animal Environment and Health, Swedish University of Agricultural Sciences, Uppsala, Sweden

**Keywords:** thermal tolerance, climate warming, brain function, neural failure, electroencephalogram (EEG), cardioventilatory performance

## Abstract

As an effect of climate change, heatwaves pose an increasingly more frequent and severe threat to fish populations. Yet, the physiological mechanisms underlying thermal tolerance in fish remain unclear. One hypothesis is that thermal tolerance may be limited by neural failure at high temperatures. Here, we used an electrophysiological approach to test this by assessing the relationship between brain function, determined via recordings of visually evoked responses (VERs) on the electroencephalogram (EEG), and cardioventilatory performance, determined via recordings of ventilatory electromyography (EMG) and electrocardiogram (ECG), in adult rainbow trout (*Oncorhynchus mykiss*) exposed to a critical thermal maximum (CT_max_) protocol. Our results show that normal brain function is preserved at moderate to high temperatures; however, at CT_max_, the fish exhibited loss of VERs, indicating brain dysfunction associated with insensibility. This suggests a strong link between neural failure and upper thermal tolerance in fish. Although heart and ventilatory rates increased with warming, heart rate significantly declined at CT_max_. Interestingly, ventilation rate remained high even at extreme temperatures and at CT_max_, indicating that neural ventilatory drive was maintained across thermal extremes. The factors underlying thermally induced neural failure and its implications for fish in a warming world require further investigation.

## 1. Introduction

Anthropogenic warming of aquatic ecosystems and the increasing prevalence of transient heatwaves pose serious threats to fish at individual and population levels [1]. Indeed, ocean warming has been linked to shifts in the spatial distribution of fishes [2,3] and correlates with species-specific thermal tolerance limits [4,5]. Heatwaves, in particular, may exert a strong selective force by causing mass mortality events [6–8]. However, the physiological mechanisms that underlie thermal tolerance in fish are still not fully understood.

As some fish suffer from insufficient oxygen delivery to the heart at high temperatures, thermal tolerance in fish is often linked to compromised cardiac function. Yet, many other physiological organs and cellular level functions are also impaired at increased water temperature, but the role of some of these factors in dictating the upper thermal tolerance limit remains elusive (see reviews by [9–13]). For example, the role of neural failure in defining the acute upper thermal tolerance limit remains poorly explored. It has been shown that localized cerebral heating via an implanted heating coil in goldfish (*Carassius auratus*) induces behavioural disturbances and loss of equilibrium (LOE, which is used as an indicator for the critical thermal maximum, CT_max_) at temperatures comparable to whole-body warming protocols [14]. Additionally, cooling the brain by 2–6°C with a mounted brain cooler raised the CT_max_ of Atlantic cod (*Gadus morhua*) by 0.5–0.7°C during whole-animal acute warming [15]. These findings suggest that temperature-induced LOE may stem from central nervous system (CNS) failure. Additionally, neural activity in locomotor brain regions and neural responses to visual stimuli in agar-embedded zebrafish larvae are significantly suppressed at temperatures approaching CT_max_ [16]. However, the impacts of acute warming on brain function have not been directly evaluated in adult fish. A promising method to assess brain function in unrestrained fish is to assess the presence or absence of visually evoked responses (VERs) in the beta waves of the electroencephalogram (EEG). Beta waves in the EEG are associated with wakefulness, alertness and consciousness. Within this activity, VERs represent measurable changes in brain electrical potential triggered by visual stimuli (e.g. a flashing light), producing a distinct waveform in the EEG milliseconds after the stimulus in conscious animals [17,18]. The absence of VERs has recently been established as a clear and objective indicator of brain dysfunction and, consequently, loss of sensibility in various fish species [19–22] (see review by [23]). Temperature-induced declines in neural function seem to coincide with reductions in ventilation and/or heart rate in adult fishes and other aquatic ectothermic organisms at temperatures approaching CT_max_ (for reviews, see [9,13]). This may reflect that both ventilatory and cardiovascular functions are regulated by the central and autonomic nervous systems [24,25] (see review by [26]), and thus, cardioventilatory activity is possibly compromised by impaired neural function (likely in conjunction with other limiting factors discussed elsewhere, e.g. see [9,13,27]). Nevertheless, the relationship between brain function, cardioventilatory function and CT_max_ remains unexplored in free-swimming adult fish.

In this study, we used electrophysiological measurements to assess the effects of acute warming on brain function and cardioventilatory performance in adult rainbow trout (*Oncorhynchus mykiss*). We used non-invasive techniques to determine the presence or absence of VERs on the EEG. We also assessed cardioventilatory performance via water electrode recordings of ventilatory electromyography (EMG) and electrocardiogram (ECG). We hypothesized that if acute warming tolerance (i.e. CT_max_) is dictated by neural failure and brain dysfunction, then neural activity in the beta wave frequency range (12−32 Hz), associated with wakefulness, would decline, and that VERs would disappear at the point when fish lose equilibrium and reach CT_max_. Additionally, we hypothesized that this loss in brain function would align with a decline in cardioventilatory performance.

## 2. Methods

### (a) Study animals

Rainbow trout of mixed sexes (*n* = 18; body mass = 978 ± 35 g; fork length = 402 ± 5 mm; Fulton’s condition factor = 1.5 ± 0.04, thus indicative of fish in a healthy condition; see [28]) were obtained from a local fish farm (Vänneåns Fiskodling AB, Knäred, Sweden). Fish were held in a holding tank continuously supplied with 10°C aerated, filtered and UV-sterilized fresh water. A photoperiod of 12 : 12 h, light : dark was maintained, and fish were fed with commercial food pellets twice weekly, but fasted for 3 days prior to experiments. All fish were acclimatized for at least three weeks to these conditions prior to the experiments.

### (b) Instrumentation and experimental protocol

**Figure 1.**
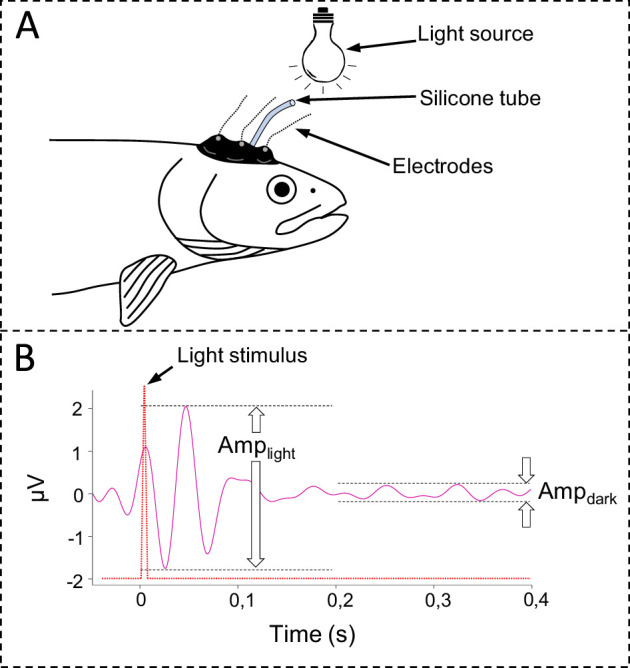
Recordings of the visually evoked responses (VERs) in rainbow trout (*Oncorhynchus mykiss*). (A) A silicone suction cup with integrated electrodes was positioned over the optic lobe to record EEG activity. (B) A representative EEG recording depicting a VER following a 10 ms light stimulus (red dashed line). VER amplitude was calculated as the difference in the brain electrical potential following the light stimulus (Amp_light_) and during the dark cycle (Amp_dark_). VERs were assessed by averaging the beta wave response to 120 light flashes over a 1 min period, allowing the determination of the presence or absence of a stimulus-locked neural response.

**Figure 2.**
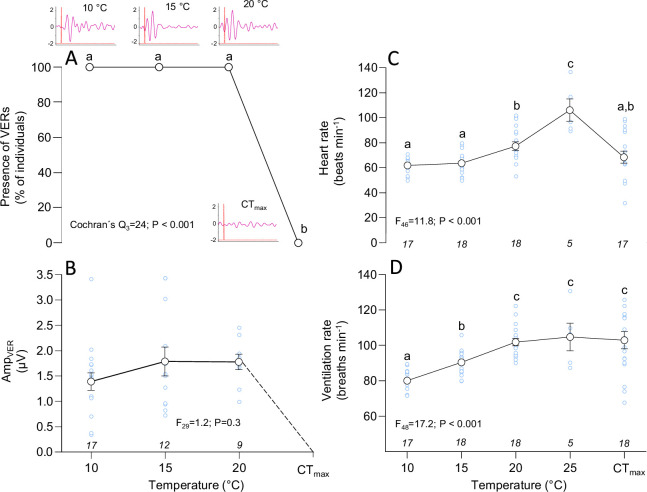
Effect of acute warming on brain function and cardioventilatory activity in rainbow trout (*O. mykiss*). (A) The proportion of detected VERs, with inset example depictions of VERs across the temperature range, and (B) the relative change in VER amplitude (AMP_VER_). Depicted average values only include values from individual fish in which VERs could be detected, as indicated by the sample size (n) at the bottom of the panel. (C,D) Changes in heart rate and ventilation rate, respectively, during acute warming and at CT_max_, with sample sizes (n) indicated in each panel. The results from Cochran’s Q test or the linear mixed models are depicted in each panel. Dissimilar letters denote statistically significant (*p* < 0.05) differences for each variable between temperatures or CT_max_.

Individual fish were lightly anaesthetized in 10°C water containing 75 mg l^−1^ MS-222 (Sigma-Aldrich Inc., St Louis, Missouri, USA) buffered with 150 mg l^−1^ NaHCO_3_. Thereafter, a silicone suction cup with integrated cutaneous electrodes to record EEG (see details below, figure 1A) was placed on the skull above the optic lobe (see [21,22]). The fish was then transferred to an experimental chamber (approx. 16 l, length: 535 mm, width: 198 mm, height: 148 mm) supplied with flow-through, aerated water at 10°C, in which the fish was fully immersed and unrestrained. To shield the individual from external disturbances, the experimental chamber was covered with a glass lid and black plastic drapes, while the room was kept dark. Recordings of heart rate, ventilation and EEG in response to light stimuli (i.e. to determine VERs, see details below) were initiated immediately following placement in the chamber and were maintained continuously throughout the entire experimental protocol. These recordings allowed for verification of signal quality, correct electrode placement, recovery from anaesthesia and the determination of physiological responses leading up to and following CT_max_. Fish were allowed to recover for 30 min prior to the thermal challenge, which consisted of increasing the water temperature at a rate of approximately 0.25 °C min^−1^ using a thermostat-controlled 6 kW heating element, which heated the water in an external tank connected in series with the experimental chamber. When the temperature had reached 15, 20 or 25°C, the temperature was maintained for 20 min, allowing for thermal equilibration of body tissues [29]. Warming increased until the fish lost equilibrium, which was visually determined by the experimenter, and the specific temperature at which this occurred was determined as CT_max_ [30]. If the suction cup was dislodged during the protocol, e.g. due to stress-induced agitation during the thermal ramping (which is reflected in the varying sample sizes across temperatures, see figure 2), immediate re-attachment was attempted on the fish inside the experimental chamber. Immediate re-attachment involved briefly and gently manually restraining the fish (typically for <5 s) within the darkened experimental chamber during the intermittent light flashes to re-attach the suction cup. This intervention did not alter the light settings or affect the VERs of the fish, as these were assessed during subsequent epochs after the fish had been left undisturbed in the chamber. If unsuccessful, re-attachment was done immediately following CT_max_. Water oxygen levels in the experimental chamber were maintained above 92% air saturation throughout the thermal challenge. At CT_max_, fish were immediately removed from the experimental chamber and euthanized with a percussive blow to the head, after which body mass and fork length were recorded.

### (c) Neural activity recording and analyses

All recording equipment was connected to a PowerLab (AD Instruments, Australia) and a computer running Labchart Pro software (v. 7.3.8, ADInstruments, Australia) for data collection. EEG was recorded using the non-invasive recording technique described in detail previously [21,22]. Briefly, silver chloride electrodes connected to 1.5 mm shielded copper wires were mounted in a custom-made silicone suction cup fitted with a 2 mm silicone tube connected to a peristaltic pump to attach the silicone cup on the head of the fish with negative (suction) pressure (figure 1A). The electrodes were connected to a BioAmp (AD Instruments, Australia), and the raw EEG signal was optimized by filtering the signal within the 200 μV sensitivity range using a 60 Hz low-pass filter, a 0.1 Hz high-pass filter and a 50 Hz notch filter. Light stimuli were generated by a custom-built stroboscopic LED array mounted above the experimental chamber, delivering 10 ms flashes at approximately 100 lx and 2 Hz, with each flash separated by a dark phase at or near 0 lx. The light stimuli were detected and recorded using a custom-built light sensor connected to the PowerLab unit.

EEG recordings were subsequently processed offline using a bandpass filter (12–32 Hz) to separate out the beta wave frequency range [22]. A 400 ms segment of beta waves was obtained after each light flash, and 120 consecutive segments (i.e. 1 min) were compiled using the Scope view function in LabChart to create an averaged response to the light stimulus at each temperature (see [21,22]). Segments with signal amplitudes exceeding 10 μV (indicative of motion artifacts) were excluded, and averaged responses containing <50% of total segments were considered unreliable and omitted from the analysis [22]. A VER was defined as ‘present’ if the magnitude of change of the average EEG amplitude following the light stimulus (Amp_light_, 0−200 ms) was at least two times higher than the average baseline amplitude during the dark cycle (Amp_dark_, 200−400 ms, figure 1B). The amplitude of a VER (Amp_VER_) was calculated as the difference in the brain electrical potential between Amp_light_ and Amp_dark_ (figure 1B) and was taken during the final minute of the thermal equilibration periods for each temperature increment (i.e. at 10, 15 and 20°C) and at CT_max_. No further analyses of spontaneous EEG features were conducted, as such signals are highly susceptible to noise from movement and physiological artefacts, making them unreliable for assessing neural function in unrestrained fish under these experimental conditions.

### (d) Cardioventilatory recording and analyses

Bioelectrical potentials generated from the heart and ventilation (i.e. ECG and EMG) were obtained with electrodes in the water in the experimental chamber [31,32]. The raw ECG signal from the electrodes was amplified using a BioAmp (AD Instruments, Australia), and the signal was optimized within the 500 μV sensitivity range, and using a 5 kHz low-pass filter, a 1 kHz high-pass filter and a 50 Hz notch filter. This signal was further filtered to separate out the R wave of the ECG (bandpass filter: 50−10 Hz), which represents the ventricle contraction and ventilatory activity (bandpass filter: 3−1 Hz). Heart and ventilation rates were calculated by counting the R wave peaks and ventilation cycles per minute, respectively. The effects of temperature on heart rate and ventilation were recorded during the final minute of the thermal equilibration periods for each temperature increment (i.e. 10, 15 and 20°C) and at CT_max_.

### (e) Statistics

Statistical analyses were performed using IBM SPSS Statistics 28 software, and statistical significance was accepted at *p* ≤ 0.05. Values are presented as mean ± s.e.m. The presence or absence of VERs across the different temperature levels (i.e. 10, 15, 20°C and at CT_max_) was determined by a Cochran’s Q test. Only fish for which VERs could be detected were included in this analysis. Significant main effects were further evaluated with pairwise comparisons across temperatures with multiple McNemar tests with Bonferroni corrections. Linear mixed models were performed with individual values for heart rate and ventilation rate as the dependent variables, and temperature levels (10, 15, 20, 25°C and CT_max_) as the independent repeated measures variable. Significant main effects were further evaluated with Bonferroni-corrected pairwise comparisons across temperatures.

## 3. Results and discussion

In accordance with our hypothesis, VERs were lost when fish reached CT_max_ (25.6 ± 0.3°C), a thermal threshold consistent with previous studies using a similar strain of rainbow trout acclimated at 10°C [11,33]. This illustrates brain dysfunction at these extreme temperatures. While heart rate declined at CT_max_, the ventilation rate remained elevated. Thus, our findings indicate that at CT_max_ some brain functions (visual input processing in the visual cortex), but not all (i.e. neural ventilatory control pathways), are impaired in rainbow trout.

In all individuals in which a reliable EEG could be recorded, VERs were present across all temperatures up to 20°C (figure 2A). At 10°C, the average Amp_VERs_ was 1.4 ± 0.2 μV (329 ± 56% above baseline, figure 2B), which is similar to previous values reported in rainbow trout at 10°C [21,22]. Amp_VERs_ remained stable across temperatures at 1.8 ± 0.3 and 1.8 ± 0.2 μV (408 ± 93% and 300 ± 49% above baseline) at 15 and 20°C, respectively. However, no VERs were detected at CT_max_ in any of the assessed trout (*n* = 18; figure 2A). This provides clear evidence that brain function is severely impaired at CT_max_ in rainbow trout. Our data share similarities with the findings in zebrafish larvae by Andreassen and colleagues [16], who recorded neural activity by quantifying the frequency of calcium peaks in the medulla or the optic tectum using calcium imaging. These authors showed that the frequency of calcium peaks, and thus the spontaneous medullary neural activity and the responsiveness to visual stimuli in the optic tectum, declined at temperatures preceding CT_max_. Thus, our findings further indicate that extreme high temperatures induce neural dysfunction that causes loss of visual perception and locomotory control, which in the latter case has been indicated in other fish species [14,15]. Such dysfunction would reduce the ability of fish to escape unfavourable thermal conditions and to behaviourally thermoregulate, potentially leading to fatal consequences during a heatwave.

The underpinnings for neural failure at high temperatures in fish remain elusive. Friedlander and colleagues [14] related the behavioural impairments and CT_max_ following heating of the cerebellum in goldfish to declining interneuron activity. Indeed, neurons are optimized to a specific thermal range [34–36], and declining neural function may be caused by increased permeability and reduced stability of excitable neural membranes [16,27,37], and/or failing mitochondrial function in neural tissues at high temperatures [35]. Moreover, Andreassen *et al.* [16] demonstrated that a decrease or increase in water oxygen content led to negative or positive impacts, respectively, on neural activity, responsiveness to visual stimuli and CT_max_. This indicates that the upper thermal limit of neural function is at least partially dictated by cerebral oxygen limitation in zebrafish larvae, which rely predominately on cutaneous respiration during this life stage.

**Figure 3.**
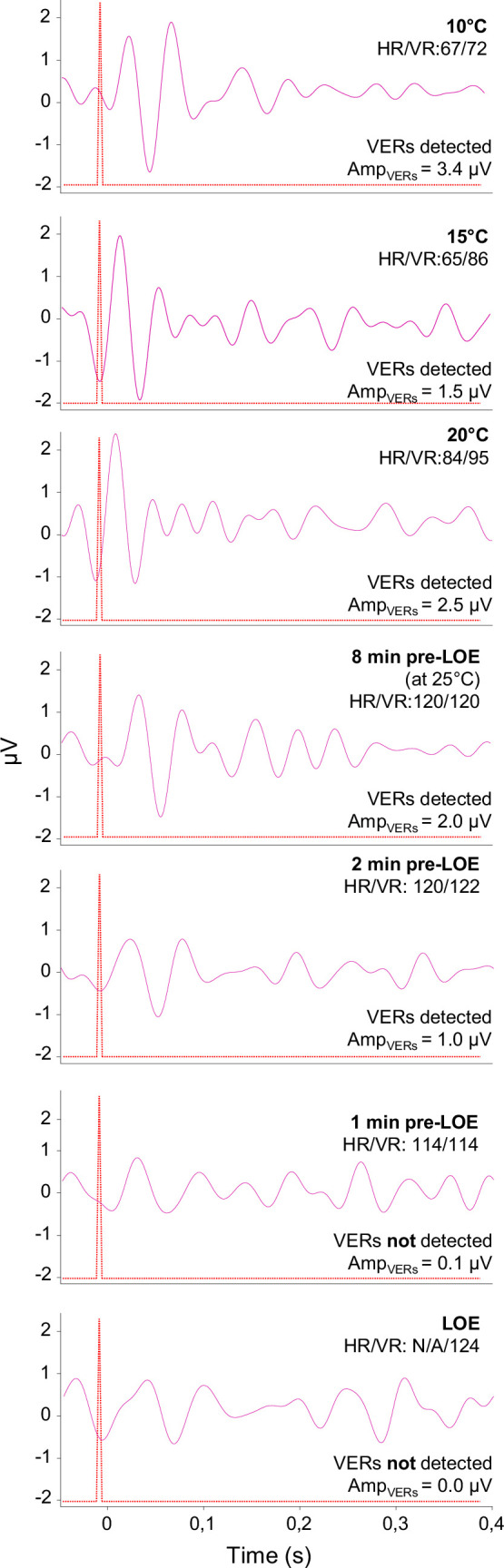
Effect of acute warming on brain function and cardioventilatory responses in rainbow trout (*O. mykiss*). The panels depict the presence or absence of VERs following a light stimulus (red dashed line), heart rate (HR) and ventilation rate (VR) at 10, 15 and 20°C, and at 25°C in the minutes prior to the onset of CT_max_, as indicated by loss of equilibrium (LOE), in one individual fish during an acute thermal exposure.

It is likely that a decline in cardiac function and thus a reduced cardiovascular oxygen transport and supply to cerebral tissues and/or locomotory muscles contributed to neural dysfunction and the onset of CT_max_, as suggested by our data and previous work on zebrafish larvae (e.g. see [16]). Indeed, consistent with previous observations in numerous fish species (summarized in [13]), both heart rate and ventilation rate increased with warming, from 62 ± 1 beats min^−1^ and 80 ± 1 breaths min^−1^ at 10°C, respectively, to a maximum of 106 ± 9 beats min^−1^ and 105 ± 8 breaths min^−1^ at 25°C, respectively (figure 2C,D). At CT_max_, however, heart rate decreased to 65 ± 5 beats min^−1^, and this reduction is consistent with previous findings showing that heart rate and cardiac function typically plateau or decline at critically high temperatures and is therefore considered a crucial factor for governing CT_max_ in salmonids and other fish species [9,11,13,16]. The decline in heart rate is likely to some extent to reflect the onset of cardiac arrhythmias as elevated temperatures, as seen previously in rainbow trout and other fish species [11] (for a review see [13]), but could also reflect an impairment of neural autonomic regulation of cardiac functions, or possibly an insensibility of the fish to stressful stimuli due to brain dysfunction. Interestingly, the ventilation rate remained elevated at CT_max_ in trout (103 ± 5 breaths min^−1^), as also observed in our previous work in this strain [11]. Moreover, successful continuous recordings of VERs in the minutes preceding CT_max_ in one individual fish indicate that brain function was sustained 2 min prior to CT_max_, at which the VERs disappeared (figure 3). While both heart rate and ventilation rate declined slightly 1 min prior to CT_max_, at which heart rate was undetectable due to signal disturbances, ventilation rate remained high at CT_max_ in this individual. While intriguing, we caution against a generalizable interpretation of these observations stemming from a single individual fish. Unfortunately, similar analyses for the remaining individuals were not possible due to EEG cup dislodgement and/or heart and ventilation signal disturbances due to fish movement prior to CT_max_. Collectively, our data show that neurally mediated motor output to respiratory muscles from respiratory control centres located in the brainstem [24,25] seemingly remained intact, even while visual input processing in the optic lobes and locomotory control of body posture failed at high temperatures, suggesting a divergence in the thermal sensitivity of central nervous and/or efferent neural pathways in rainbow trout.

In conclusion, our findings demonstrate that the warming tolerance of rainbow trout is linked to brain dysfunction associated with insensibility and a reduction in heart rate at CT_max_, while ventilation rate remains elevated, indicating intact ventilatory regulation at CT_max_. Further exploration of the causal factors underlying thermally induced brain dysfunction in fish is warranted to enhance our understanding of how warming environments associated with climate change affect fish survival.

## Data Availability

The original data from which the results of the current manuscript are based are available from the Dryad Digital Repository [38].
